# Analytic measures for quantification of arousal from spontaneous skin conductance fluctuations

**DOI:** 10.1016/j.ijpsycho.2010.01.011

**Published:** 2010-04

**Authors:** Dominik R. Bach, Karl J. Friston, Raymond J. Dolan

**Affiliations:** Wellcome Trust Centre for Neuroimaging, University College London, 12 Queen Square, London WC1N 3BG, United Kingdom

**Keywords:** Skin conductance (SCR), Galvanic skin response (GSR), Electrodermal activity (EDA), Linear time-invariant filter (LTI), Area under the curve (AUC)

## Abstract

Autonomic arousal is often indexed by spontaneous fluctuations in skin conductance. Here, we derive a simple measure of sympathetic arousal, using a convolution model of how sudomotor bursting causes fluctuations in skin conductivity. Under this model, the time-integral of measured conductance is proportional to the frequency and amplitude of sudomotor bursts. We demonstrate the validity of this measure in relation to finite impulse response models, and show that it is a better predictor of autonomic arousal, relative to conventional measures.

Emotional arousal is often indexed by measuring skin conductance [*SC*]. *SC* changes are mainly dependent on the activity of sweat glands innervated by the sympathetic branch of the autonomic nervous system [ANS]. The most frequently used measures of tonic ANS activity are the number of spontaneous fluctuations [*SF*] in skin conductance, the amplitude of these fluctuations, and the overall level of the skin conductance [*SCL*] (see for an overview Boucsein, 1992).

Many methods for *SF* detection require visual inspection, and introduce a subjective element into the analysis. Existing computational strategies for *SF* mandate a search for peaks in the signal, and the exclusion of peaks that do not to resemble a canonical response. These exclusions are based either on a heuristic of estimated rise and decay time or, alternatively, by fitting polynomials to the response and excluding those that cannot be fitted (see e.g. Trosiener and Kayser, 1993; Storm et al., 2000 for examples of such algorithms). The particular analytical form, and the parameters used, in these approaches are optimised to reflect a reference standard that is derived from visual scoring and not from theory. This means that although a relationship exists between the number, or amplitude, of *SF* and sympathetic arousal, the precise relationship between the number of *detected SF* and sympathetic arousal is difficult to describe in analytic terms. These shortcomings suggest a need for a measure that provides a more precise mapping between an analytical form and the physiological variable under study. This approach differs from signal deconvolution strategies (Alexander et al., 2005; Benedek and Kaernbach, 2009) that try to recover the sudomotor nerve activity time series but do not quantify sympathetic arousal.

Here, we develop a simple measure of autonomic arousal based on linear time-invariant [LTI] assumptions and validate it on a previously published dataset in the context of anxiety aroused by public speaking anticipation. Software implementation of this method is freely available as function scr_sf_auc.m within the previously published software suite *SCRalyze* (Bach et al., 2009; 2010) obtainable from http://scralyze.sourceforge.net under the *GNU General Public License*.

## 1 Theoretical overview

*SF* are caused by sporadic and spontaneous (i.e. unrelated to experimentally presented events) activity of the sudomotor nerve (Boucsein, 1992). Spontaneous firing occurs in short bursts with a duration of around 500–1000 ms, separated by longer intervals, and is followed by opening of sweat glands (Macefield and Wallin, 1996; Nishiyama et al., 2001). The number of sweat glands recruited is linearly related to the amplitude of a firing burst (Nishiyama et al., 2001). Consequently, it is plausible to assume that the amplitude of an *SF* is linearly related to the amplitude of the firing burst. Further, it appears from previous research that both the number and the amplitude of bursts reflect sympathetic arousal.

It is biophysically plausible that the measured signal has some relationship with sudomotor nerve [*SN*] firing and reasonable to assume that this relation is constant (that is, time-invariant), and that two subsequent responses will build up in a linear fashion. Under these LTI assumptions, and in the absence of noise, it is easy to see that the time-integral (or area under the curve) of an *SC* time series is simply the *SCL*, plus the number of responses *n*, scaled by their amplitude *a*, and multiplied by a constant *c*. This constant is the time-integral of a single response to an input of unit amplitude (i.e., the response function; *RF*).

(1) $$\begin{array}{c} SC\left(t\right)=SCL+SN\left(t\right)⊗RF\left(t\right)\Rightarrow \\ \int SC\left(t\right)\mathrm{d}t=SCL+c\sum_{i=1}^{n}a_{i}=SCL+cn\overset{―}{a}\\ c=\int RF\left(t\right)\mathrm{d}t\\ SN\left(t\right)=\sum_{i=1}^{n}a_{i}\delta \left(t-T_{i}\right) \end{array}$$

where we describe *SN* as a Dirac delta function, and where *ā* is the mean amplitude of spontaneous fluctuations occurring at times *T*_*i*_: *i* ∊ 1,…,*n*.^2^ The *SCL*-corrected time-integral, or area under curve [*AUC*], which is simple to compute, should therefore reflect the number and amplitude of sudomotor bursts and the status of the sympathetic nervous system. In reality, LTI assumptions are unlikely to be met completely. However, we can posit that

(2) $$\int SC\left(t\right)\mathrm{d}t=SCL+cn\overset{―}{a}+e$$

where *e* denotes some error that absorbs random fluctuations and any violations of time-invariance and linearity assumptions. This is the model that we seek to validate in this paper. Note that a similar measure has been used in applied psychophysiology to quantify arousal during anaesthesia (Ledowski et al., 2007), but has not been formally derived or validated. Here, we provide a measure for the integrated time series, corrected for *SCL* by subtracting the lowest signal value, which we refer to as the area under the curve.

An alternative measure that has been previously used is the spectral power of the signal (Shimomura et al., 2008). If we regard the skin conductance time series *SC* as a convolution of a time series of sudomotor firing bursts *SN* with a time-invariant response function

(3) $$\begin{array}{c} SC\left(t\right)-SCL=SN\left(t\right)⊗RF\left(t\right)\Rightarrow \\ FT\left(SC-SCL\right)=FT\left(SN\right)FT\left(RF\right) \end{array}$$

then, according to the convolution theorem, we can write the Fourier transform [*FT*] of the skin conductance time series as a product of the *FT* of nerve firing and response function. This is the same as Eq. (1) but now we are treating the sudomotor input as a continuous times series (as opposed to a series of discrete events). The overall spectral power of the (*SCL*-corrected) skin conductance time series will vary with the amplitude of sudomotor firing, while the frequency of sudomotor bursts will influence low frequencies of the spectral power (because the inter-burst interval determines the lower frequencies): an increase in the (low) frequency of bursts will shift the frequency of spectral power in lower ranges. More formally, for a rectangular (sudomotor pulse) wave of duration *d*, occurring every 1/*n* seconds (i.e., a burst frequency of *n*), the Fourier coefficients are:

(4) $$FT{\left(SN\right)}_{i}=\frac{\sin\left(i\pi nd\right)}{i\pi }\Rightarrow \frac{\partial FT_{i}}{\partial n}=\mathrm{d}\cos\left(i\pi nd\right)\text{.}$$

This simply says that the change in the Fourier coefficients with burst frequency is greatest at low frequencies (low *i*) because cos(*iπnd*) decreases with increasing *i*, given that *nd* < 1. This is why it has been proposed previously to quantify sympathetic arousal by integrating the spectral power of the SC signal over low frequencies (Shimomura et al., 2008). However, Eq. (4) describes the power spectrum of the (unknown) *SN* and does not directly apply to the *SC* power spectrum. In fact, Eq. (3) means that the burst frequency will have its greatest impact on spectral power of the *SC* when it matches the peak frequencies of the response function. Therefore, the *SC* power spectrum captures the frequency overlap between the response function and sudomotor firing, but not the sudomotor firing itself.

If the spectral power of the response function is known, it is possible to recover the firing frequency, or even the time series of sudomotor firing using

(5) $$SN=FT^{-1}\left(\frac{FT\left(SC-SCL\right)}{FT\left(RF\right)}\right)\text{.}$$

However, noise and response variability render Eq. (5) useless for practical purposes (see Alexander et al., 2005 for a similar deconvolution approach in the time domain that does not account for noise). Although classical methods are available for deconvolution with known noise spectra (e.g., Wiener deconvolution and related approaches), we pursue the time domain formulation in Eq. (2), because its application does not rely on knowing the noise spectrum.

## 2 Data

We analysed a dataset published previously (Bach and Erdmann, 2007, 2008) that contained 1153 *SF* from four measurements of 40 healthy male university students (18–35 years) that were subjected to a public speaking anticipation paradigm after giving informed consent. The main focus of this paper was the interaction of habitual and situational symptom focusing, operationalised as attention towards neck muscle tension. The main experimental manipulation had no effect on indices of skin conductance such that data from the different experimental groups were combined for the present analysis, where we focus on the effect of the public speaking treatment. There were two baseline measurements, one measurement after announcement of a public speech, and another measurement after announcement of the speech topic. This allowed us to separate the effects of anxiety and cognitive effort.

After skin cleansing with propanol, skin conductance was recorded on thenar/hypothenar of the non-dominant hand using 8 mm Ag/AgCl cup electrodes (Coulbourn, Whitehall PA, USA) and 0.5%-NaCl electrode gel (Par, Berlin, Germany). 0.5 V constant voltage was provided by a S77-21 coupler (Coulbourn). The signal was band pass filtered (0.015 and 5 Hz), digitally converted with 10 Hz sampling rate (DI-205, Dataq, Akron OH, USA) and recorded (Windaq, Dataq). Each of four measurements lasted 120 s. The middle 60 s were analysed using a semi-automatic method (Event Detection and Analysis, Trosiener and Kayser, 1993) with a threshold of 0.025 μS. Note that this analysis had been done in the context of the original experiment (Bach and Erdmann, 2007), before the present method was developed, such that it can be regarded as unbiased.

Data analysis was carried out in Matlab (7.4, MathWorks, Natick MA, USA) using custom code that is available from the authors: this returns

(6) $$AUC=\int SC\left(t\right)\mathrm{d}t-SCL=cn\overset{―}{a}+e$$

from Eq. (1). After importing 60 s segments of *SC* into Matlab, no further signal conditioning was applied.

## 3 Results

**Fig. 1:**
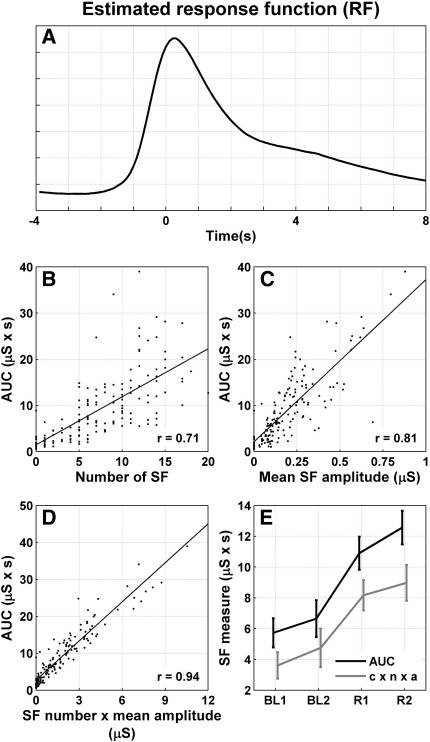
A: The response function estimated across participants with an uninformed finite impulse response model that deconvolves potentially overlapping responses. The time at 0 s corresponds to peak time as detected by semi-automated conventional analysis. B–D: Correlation of the *AUC* with conventional *SF* measures, derived from semi-automated conventional analysis. All correlations were significant (*p* < 0.0001). E: *AUC* (black) and number × mean response amplitude (multiplied with the scaling constant *c*) predict autonomic arousal in a public speaking anticipation experiment, but *AUC* is a better predictor (see text for statistical inference). BL1 and BL2: baseline measurements. R1: measurement after announcement of a public speech. R2: measurement after announcement of the speech topic. Values are shown as mean and standard error.

First, we quantified the validity of our linear time-invariant (LTI) assumptions (implicit in Eq. (1)) by ensuring we could account for the majority of observed variance with a simple LTI convolution model. To deconvolve potentially overlapping *SC* responses, we used least-squares deconvolution under an uninformed finite impulse response function model (Bach et al., 2009), consisting of 120 delta functions (or stick functions), one for each datapoint over a time window of (− 4, 8) s. We assumed a sudomotor input of fixed amplitude occurring before any *SF* peaks as identified by a semi-automated analysis. The estimated finite impulse response function from fitting this LTI model to the entire dataset is depicted in Fig. 1A.

This response function was then applied once to each peak in the entire dataset, and the amplitude of each peak was estimated. This model could explain 85.4% of the observed variance, thus suggesting that the time-invariance assumption is largely met.

We then sought to validate Eq. (6) which implies that there is a close relation between the number and amplitude (*n* × ā) of *SF* from conventional analysis and the new *AUC* measure. Also, it predicts that the constant of proportionality between the two measures equals the time-integral of the finite impulse *RF*.

Fig. 1B–D depicts the correlations of *AUC* with the number of responses, mean amplitude or responses, and number × mean amplitude (*n* × ā). The latter shows about 89% shared variance, suggesting a high degree of convergent validity between the conventional and *AUC* measures. The regression slope when regressing *AUC* on *n* × ā was 3.53 s. This is remarkably similar to the integral of the impulse response function; *c* = 3.61 s, based on the finite impulse response function estimated above (see Eq. (6)). This provides evidence for the validity of LTI assumptions. Note that the intercept was positive, such that the observed *AUC* takes an above zero value when number × mean amplitude is zero.

In order to render the two measures directly comparable, we multiplied the conventionally derived amplitude × number of responses with the constant *c* such that they were brought to the same units and scale. In a 2 (treatment: baseline/anticipation) × 2 (method) repeated measures ANOVA, both the conventionally derived measure (*F*_1, 39_ = 29.9; *p* < 0.0001) and the *AUC* (*F*_1, 39_ = 41.7; *p* < 0.0001) could predict autonomic arousal in the public speaking paradigm, but *AUC* made the more precise prediction as shown in the treatment × method interaction (*F*_1, 39_ = 42.3; *p* < 0.0001), as shown in Fig. 1E.

We then classified each epoch from each participant as either baseline or anticipation and tried to predict these categories, across all participants, from amplitude × number of responses and from *AUC*. Again, *AUC* was a better predictor (*r* = 0.38) than amplitude × number of responses (*r* = 0.30). Thus, *AUC* explained a higher proportion of variance in the categories. For this difference, we computed an *F*- and its associated *p*-value as (variance explained by *AUC* − variance explained by conventional analysis) / residual variance, corrected by the respective degrees of freedom (*F*_1, 159_ = 8.7; *p* < 0.005).

## 4 Discussion

In this paper, we derive a summary statistic for sympathetic arousal as indexed by spontaneous fluctuations (*SF*) of the skin conductance. We show that it is possible to do so in a purely analytic fashion, without ad-hoc adjustments and complicated algorithms or visual control. The statistic we derive, namely the area under the curve *AUC*, or skin conductance level (*SCL*)-corrected time-integral, is closely related to number and amplitude of *SF* as scored by an independent, conventional method. It predicts experimentally manipulated autonomic arousal in a public speaking paradigm and is a significantly better predictor than the amplitude and number of *SF* estimated conventionally and may therefore provide a less noisy estimate of sudomotor activity. This is not surprising, as we also show that *SF*s seem to be largely time-invariant and only differ in amplitude, rather than in their shape. In fact, our model is parsimonious as it uses only one response function for the whole sample and will therefore underestimate the explainable variance, but still can explain more than 80%. This is higher than for event-related skin conductance responses (*SCR*), where explained variance using one response function across individuals is typically estimated at around 50% (Bach et al., 2010). Note, however, that in the analysis of *SCR*, *SF* constitute noise and contribute to residual within-subject variance, whereas in the present approach, they are the responses of interest. As a limitation of our approach, we note that the conventionally derived *number* of responses is a better predictor of autonomic arousal than the number × mean amplitude. In other words, although our *AUC* measure provides a less noisy quantification of combined response number and amplitude than conventional analysis, this might not be the optimal quantification of autonomic arousal. Future work will investigate methods to recover the number of responses independent from their amplitude.

In summary, we show that the time-integral of the skin conductance time series provides a simple quantification for autonomic arousal that is computationally inexpensive and requires no subjective element. This approach could in theory be extended to quantify event-related (evoked and anticipatory) responses, although here one might be more interested in separating event-related from spontaneous responses which requires some regularisation of the possible response, e.g. in a general linear convolution model [GLM] (Bach et al., 2009).

## Footnotes

2 We chose to use continuous representation here, but all equations hold for discrete data by replacing integrals with sums.
